# Microbial community in alfalfa rhizosphere in response to rhizobial inoculation under increased concentrations of potentially toxic elements

**DOI:** 10.3389/fpls.2026.1800160

**Published:** 2026-06-16

**Authors:** Mila Pešić, Biljana Sikirić, Vesna Mrvić, Uroš Buzurović, Sonja Tošić Jojević, Snežana Andjelković, Olivera Stajković-Srbinović

**Affiliations:** 1 Institute of Soil Science, Belgrade, Serbia; 2 Institute for Forage Crops Kruševac, Kruševac, Serbia

**Keywords:** alfalfa, heavy metals, microbial communities, plant growth-promoting bacteria, rhizobia, rhizosphere

## Abstract

**Introduction:**

The rhizosphere is a very active region containing a large number of microorganisms involved in complex biological and ecological processes. The microorganisms can improve the soil conditions, promote plant growth, and alleviate stress in plants under heavy metal contamination. Rhizobial bacterium (*Ensifer meliloti*) forms endosymbiosis with alfalfa (*Medicago sativa* L.), providing nitrogen to the plant, and can mitigate the effects of different stress factors.

**Methods:**

This study aimed to evaluate the effects of rhizobial inoculation on alfalfa growth and rhizosphere microbiological properties in soils with increased nickel (Ni) concentrations during 2 years and across seasons. Two locations with different heavy metal concentrations, lower and higher, mainly Ni concentrations, but also lead (Pb) and chromium (Cr); six different rhizobial inoculants; and three different sampling time points were tested. The abundance of different groups of culturable bacteria (total number of microorganisms, fungi, actinomycetes, oligonitrophiles, *Azotobacter*, and ammonifiers), as well as microbial activity, that is, basal soil respiration rates, was evaluated in the rhizosphere soil of each treatment, location, and season.

**Results and discussion:**

The inoculation in some treatments significantly increased alfalfa yield in particular cuts, depending on the location (*p* < 0.05) and treatment (*p* < 0.05), with up to 38% increase compared to the control non-inoculated plants. Inoculations also influenced the abundance of specific microbial groups and soil respiration rates. Multivariate analysis of variance revealed a significant interaction between the tested factors [rhizobial inoculation (*p* < 0.05), heavy metal concentrations (*p* < 0.05), and seasons (*p* < 0.05)] on all evaluated soil microbiological properties. Generally, the total number of microorganisms was positively influenced by inoculation and varied among different rhizobial treatments within the same field and season. The increased number of rhizosphere bacteria was positively correlated with yield in some cuts.

**Conclusion:**

The results show the potential of inoculation with particular rhizobia in the improvement of alfalfa yield in Ni-contaminated soils and overall microbiological properties and indicate the complexity of the interactions of multiple factors in the environment.

## 1 Introduction

Alfalfa (*Medicago sativa* L.) is a perennial herbaceous plant from the Fabaceae family, considered as one of the most important forage crops cultivated worldwide. It has high adaptation potential to different environments, reaching high biomass yield and quality (Putnam et al., 2014). Alfalfa forms a symbiotic relationship with soil bacteria, rhizobia, which allows them to use nitrogen from the atmosphere (Maróti and Kondorosi, 2014). *Ensifer meliloti* (*Sinorhizobium meliloti*) is one of the most common endosymbionts of the alfalfa forming root nodules, where the fixation of atmospheric nitrogen takes place. In addition, rhizobia can modulate the microbial communities in the rhizosphere of plants by increasing the abundance of beneficial bacterial genera, thus shaping the host microbiome (Huang et al., 2025). The rhizosphere refers to the region surrounding plant roots that is largely influenced by plants, with vast evidence showing that communities in the rhizosphere zone differ from those present in the bulk soil (da Cunha et al., 2024). The composition of the rhizosphere microbiome is influenced by both host plant-dependent and environmental factors (Park et al., 2023).

Heavy metals are normally present in the soil in different concentrations, but their concentrations can reach levels harmful to living organisms. Nickel is among the most common heavy metals, together with chromium, copper, and lead present in contaminated soils worldwide (Campillo-Cora et al., 2025). Although Ni is an essential element necessary in small quantities for a variety of physiological processes, it is toxic at high concentrations (Rabinovich et al., 2024). Excessive Ni in plants negatively influences seed germination and seedling growth, photosynthesis, nutrient uptake and translocation, and water relations, leading to reduced growth and yield (Hassan et al., 2019). Different bacteria can tolerate heavy metals up to a certain concentration, but some of the metals including mercury and cadmium are very toxic and cannot be tolerated even at the micromolar level (Nnaji et al., 2024). In contaminated soil, besides nitrogen fixation, rhizobia can alleviate the toxicity of trace metals by promoting plant growth and by affecting the bioavailability of metals in soils (Teng et al., 2015). Pollutants can be immobilized within the soil by plants and/or bacteria (Saharan et al., 2023; Zhakypbek et al., 2024). Some bacterial species, including rhizobia, promote the growth of various plants in metal-contaminated soil (Wani and Khan, 2013; Guo and Chi, 2013).

Soil microorganisms are crucial for soil functioning (nutrient cycling, organic matter cycling, and carbon sequestration), and microbial properties are recognized as indicators of soil health as well as heavy metal pollution. Microbial properties that serve as indicators of environmental stress include microbial activity, which can be estimated through bacterial growth, fungal growth, and soil basal respiration, as well as microbial community composition and/or diversity and microbial biomass/abundance (Campillo-Cora et al., 2025). Properties of microbial communities in the soil show diverse responses influenced by both heavy metal pollution and soil physicochemical properties (Hu et al., 2021).

The studies showed that the application of bacteria through inoculation can improve plant growth and yield, as well as alter soil microbial community composition, recruiting more beneficial rhizosphere bacteria (Di Salvo et al., 2018; Huang et al., 2024). Duan et al. (2022) showed that inoculation with *E. meliloti* had positive effects on plant growth and alleviation of Cu-induced stress and microbial properties. Inoculation of alfalfa with rhizobia (alone or co-inoculated with other microorganisms) can increase biomass production and resistance to heavy metals by increasing the antioxidant capacity and enriching the bacterial taxa beneficial for nutrient uptake (Wang et al., 2021). Enhanced growth and abundance of beneficial bacterial genera, such as *Bacillus* and *Rhizobium*, were also documented when soybean was inoculated with *Rhizobium subbaraonis* TY15 (Huang et al., 2025). The capability of *Sinorhizobium* to absorb different heavy metals into the cells or adsorb them on the cell surface has been documented (Zribi et al., 2011). Edulamudi et al. (2021) showed the potential of rhizobia to improve horse gram plant’s tolerance to Ni by increasing the total nitrogen and leghemoglobin contents, as well as accumulation of Ni inside the nodules, while reduced Ni content in the soil, due to the biosorption capacity of the rhizobial inoculant, was observed in the same study. Another study also shows the potential of rhizobial bacteria to mitigate stress caused by nitrogen deficiency or Ni toxicity by increasing the levels of non-enzymatic antioxidants (glutathione, proanthocyanidin, ascorbic acid, and flavonoids), while also improving growth and nitrogen content and reducing Ni accumulation in the roots (Yu et al., 2022).

Although there are some data regarding different potentially toxic elements (PTEs) and alfalfa, still limited information is available regarding the influence of nickel on alfalfa growth in the presence of bacterial inoculation and microbial communities (Chen et al., 2022). Our previous research reveals nickel-tolerant alfalfa-associated rhizobia with high symbiotic and plant growth-promoting potential in *in vitro* conditions (Pešić et al., 2025). Therefore, these strains were selected for further field studies to evaluate these interactions under complex environmental conditions.

The aim of the current study was to evaluate the effects of rhizobial inoculation on alfalfa growth and microbial properties in its rhizosphere zone in different soils with increased heavy metal content. We hypothesized that rhizobia with plant growth-promoting traits can improve the growth of alfalfa and shape the microbiological soil properties under heavy metal stress. For this purpose, two experimental fields were chosen, both with elevated but different Ni concentrations and one with elevated Pb and Cr concentrations, in addition to Ni. In both locations, the same field experiment was set up with alfalfa inoculated with different rhizobial strains, and the experiment was monitored for 2 years. The abundance of different groups of culturable bacteria as well as basal soil respiration rates was evaluated in the rhizosphere soil of each treatment, location, and season.

## 2 Materials and methods

### 2.1 Site description

**Figure 1:**
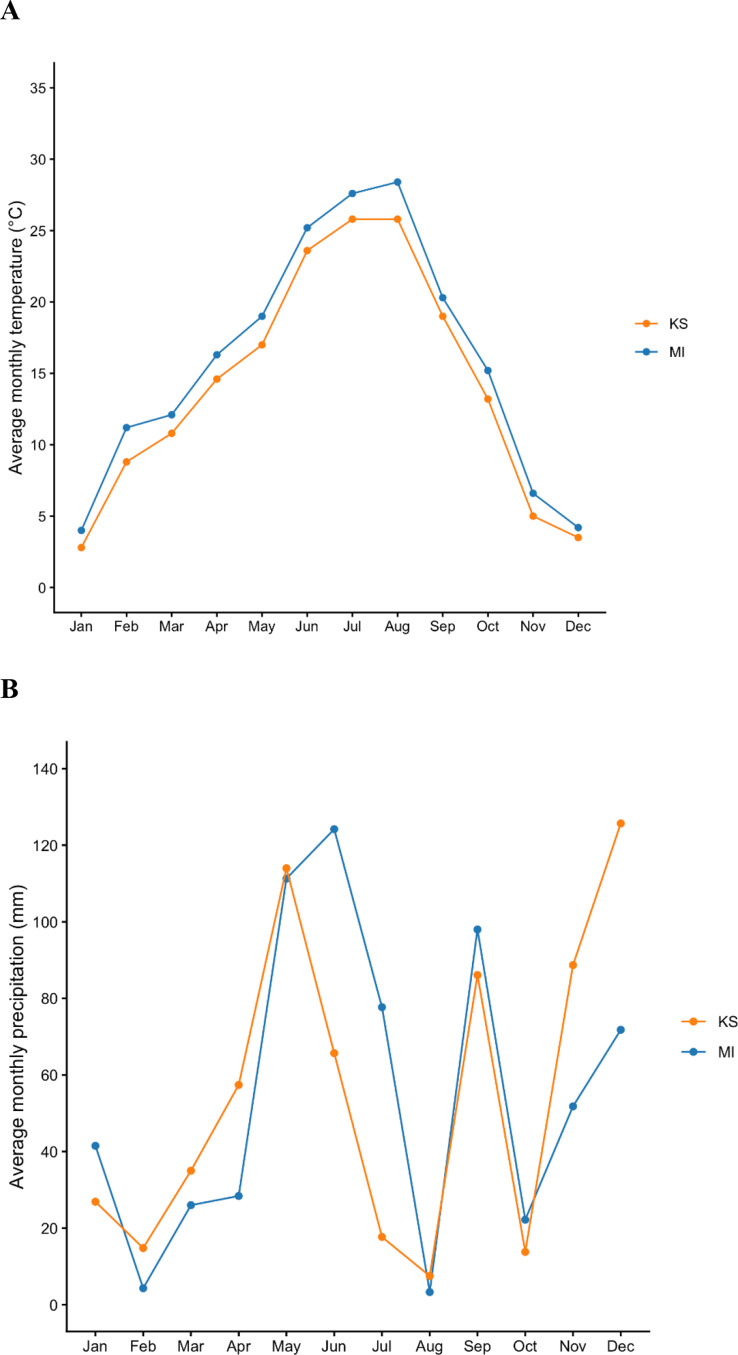
Average monthly temperatures **(A)** and precipitation **(B)** in the first year of experiment (2024).

A field experiment was conducted at two different locations in Serbia, one near Belgrade (location Mala Ivanča—labeled as MI; coordinates *x* = 469936; *y* = 4938124) and the other one near Kruševac (location Globoder—labeled as KS; *x* = 516403; *y* = 4825413). The chemical composition of the soil in the two locations is given in **Table 1**. The trace element concentrations in both locations are presented in **Table 2**. The average monthly temperatures and precipitation in Belgrade and Kruševac were obtained from the Republic Hydrometeorological Service of Serbia available at https://www.hidmet.gov.rs/, visited 22/12/2025, and were used to make a graph presented in **Figure 1**. The average annual temperature in the first year of the experiment (2024) was 15.9°C at MI and 14.2°C at the KS location (information available at https://www.hidmet.gov.rs/). The average annual precipitation in the first year of the experiment was 660.5 at MI and 653.3 mm at the KS location (information available at https://www.hidmet.gov.rs/).

**Table 1:** Physicochemical properties of the soils before the experiment was conducted.

Location	pH H_2_O	pH KCl	SOM %	Total N %	P_2_O_5_		K_2_O	Mechanical particles %			
					(mg/100 g)			Coarse sand 0.2–2 mm	Fine sand 0.02–0.2 mm	Silt 0.002–0.2 mm	Clay <0.002 mm
MI	7.36	6.36	2.62	0.12	12.24	24.62		1.7	37.8	33.0	27.5
KS	7.43	6.56	2.80	0.17	6.91	32.25		3.8	29.6	35.8	30.8

**Table 2:** Concentration of trace elements in two experimental fields before the experiment was conducted.

Trace elements (mg/kg)	As	Cd	Co	Cr	Cu	Fe	Mn	Ni	Pb	Zn
Total										
MI	6.2	0.51	17.24	46.81	20.08	n.d.	721.2	52.69*	23.46	66.22
KS	19.43	1.12	22.61	127.80*	24.16	n.d.	822.8	195.00*	130.00*	136.00
Available										
MI	0.07	0.06	0.04	bdl	2.62	26.89	19.17	1.68	2.46	1.24
KS	0.06	0.18	0.11	0.005	3.09	24.63	24.21	5.96	15.90	3.59

bdl, below the detection limit.

*Above the MAC values.

n.d. - not determined.

### 2.2 Soil chemical analyses

Soil samples were first air-dried and then ground and sieved through a 2-mm mesh. Soil pH was determined in 1 M of KCl (soil:KCl ratio = 1:2.5) or in H_2_O, while available P and K were determined by the AL method (Hans et al., 1960). Robinson’s pipette method was applied to determine the granulometric composition of the soil (Gee and Bauder, 1986). The soil total C and N were measured with an elemental CNS analyzer (Elementary Analyses system GmbH, Hanau, Germany) (Nelson and Sommers, 1996). Total C represented the organic C since the inorganic carbonates were not present in both soils. The total content of trace elements in the soil, including As, Cd, Co, Cr, Cu, Mn, Ni, Pb, and Zn, was determined using Thermo iCAP 6300 Duo (ICP-OES) following the acid digestion of the soil with HNO_3_ [ISO 22036 (2008); SRPS ISO 11466 (2004)]. The available forms of these elements were determined using Thermo iCAP 6300 Duo (ICP-OES) following extraction using diethylenetriaminepentaacetic acid (DTPA) [SRPS ISO 10694 (2005)].

### 2.3 Experimental site description and experimental setup

The field experiment was conducted in 2024 in two locations, one near Belgrade (MI) and one near Kruševac (KS), where the soil was characterized as Fluvisol (IUSS Working Group WRB, 2022). The texture of both fields is light clay, with a slightly acidic reaction. The soil organic matter (SOM)) content was 2.62% and 2.80%, total N was 0.12% and 0.17%, available P was 12.24 and 6.91 mg/100 g, and K was 24.62 and 32.25 mg/100 g in the MI and KS locations, respectively (**Table 1**).

The KS experimental field had higher concentration of all tested trace elements, with especially pronounced differences in Ni, Cr, and Pb concentrations (**Table 2**). According to Serbian official legislation (maximum allowed concentration—MAC; Official Gazette, 1994) for agricultural soils, in both experimental fields, the concentration of Ni was above the maximum allowed but below the remediation level (200 mg/kg). The concentrations of Cr and Pb in the KS location were also above the MAC. Both soils had naturally elevated levels of trace elements.

### 2.4 Field experiment setup

**Figure 2:**
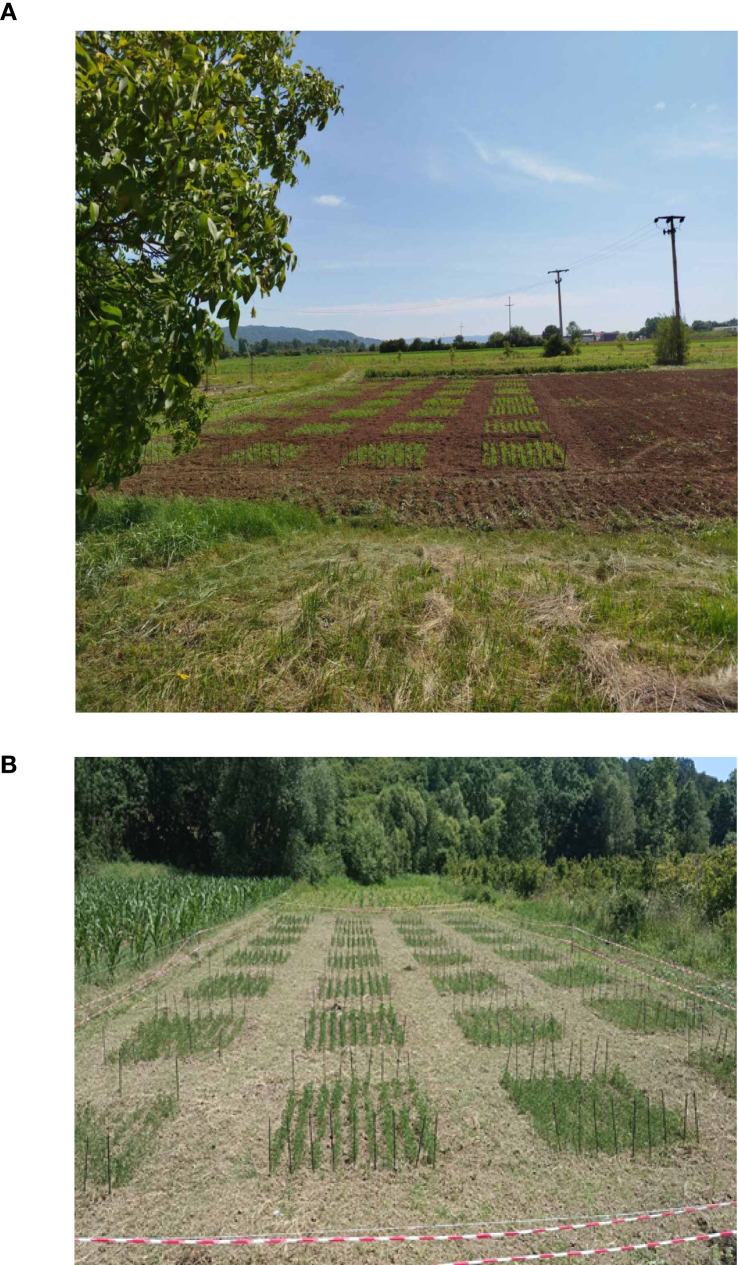
Experimental field design in KS **(A)** and MI **(B)**.

The experiment contained seven different experimental groups of plants: six inoculated with different *E. meliloti* isolates, labelled as 218, 224, G-nov, 4193cs, 217k, and 252, and one uninoculated control (control). The bacterial culture was obtained by growing the isolates in YMB at 28°C and 150 rpm for 72 h, and the culture OD_600 nm_ was adjusted to 1.00 and had 10^9^ CFU/mL. Alfalfa seeds were inoculated on the day of sowing. The experimental plots had dimensions of 1.6 m × 2 m with a 2-m space between two neighbouring squares. Inside each of the square plots, the seeds were sown in 9 rows uniformly spaced from each other, and 0.7 g of seeds were sown in each of the rows. Each of the six inoculated treatments as well as the uninoculated control was sown in four square plots (four replicates) arranged in a completely randomized design. Both experimental fields are shown in **Figure 2**.

### 2.5 Soil sampling for chemical and microbiological analyses

Soil samples were collected in the autumn of the first year (2024) of the experiment and in the spring and autumn of the second year (2025) of the experiment. Three randomly chosen plants from each square plot, with the entire root system and the soil attached to it, were extracted. Large clumps of soil adhering to the root were manually removed, while the fine soil closely attached to the root surface was collected for the subsequent analyses. Approximately the same amount of fine soil from each of the three sampled plants from each of the square plots was collected, thus making one composite sample from each of the square plots. All analyses were conducted with a minimum of three replicates. At the end of the first flowering period, three random plants were picked from each experimental square plot, and the position, appearance, and apparent number of nodules per plant from each square plot were compared across treatments. Soil samples were kept at 4 °C until used for analyses.

### 2.6 Determining the abundance of functional groups of microorganisms in the rhizosphere

In the rhizosphere soil samples, the number or the most probable number (MPN) of specific groups of microorganisms was determined by making serial dilutions of soil, inoculating appropriate media for each microbial group, and counting the specific groups of microorganisms after the period of incubation. The total number of microorganisms was determined on a medium containing soil extract and agar after 7 days of incubation at 28 °C (Stajković-Srbinović et al., 2018). The number of fungi was determined on Czapek agar after 4 days of incubation at 28 °C (Stajković-Srbinović et al., 2018), and the number of oligonitrophiles was determined after 7 days of incubation at 28 °C on Fyodorov agar. The number of actinomycetes was determined on agar plates according to Krasilnikov after 7–10 days of incubation at 28 °C (Goveradica and Jarak, 1996). The most probable numbers of *Azotobacter* and ammonifiers were determined by counting positive tubes containing liquid medium with mannitol and asparagine, respectively (Vojinović et al., 1966).

### 2.7 Determining basal soil respiration rate

The soil respiration rate was determined by incubating 30 g of soil, which was previously sieved through a 2-mm mesh sieve, in a glass inside a closed vessel at a constant temperature (28 °C) and moisture. Inside the vessel, an open bottle containing 10 mL of 0.5 M NaOH was placed. After 7 days of incubation, the amount of CO_2_ released in respiration and trapped by NaOH was determined by backtitration of the remaining OH^−^ ions by 0.05 M HCl. The amount of respired CO_2_ was calculated as μg CO_2_-C per g of soil dry weight (DW) as in Horwath and Paul (1994).

### 2.8 Alfalfa yield

To assess the effects of inoculation on alfalfa yield, the shoots of fresh alfalfa plants from each plot were cut approximately 7 cm aboveground and weighed as fresh weight at the time of rhizosphere soil sampling (last cuts in both years). Randomly collected samples of up to 1 kg from fresh alfalfa in each plot were dried at 75°C until stable weight. The ratio of dry weight to fresh weight of the sample was calculated, and the dry matter yield of alfalfa per plot was determined. The shoot dry weight of each treatment was expressed as g per square meter as the main yield parameter.

### 2.9 Statistical analysis

Multivariate analysis of variance (MANOVA) and analysis of variance (ANOVA) were conducted to examine the effect of location, rhizobial treatment, season, or their interaction on the tested parameters of microbial and chemical properties of soil and alfalfa yield. Before analysis, data were checked for normality and homogeneity using the Shapiro–Wilk and Levene’s tests, respectively, and logarithmic transformations were performed if necessary. Significant differences among the treatments were determined using Duncan’s multiple range test (*p* < 0.05). The Pearson correlation coefficient was determined between the tested parameters. The relationships between selected parameters were assessed using redundancy analysis (RDA). Structural equation modelling (SEM) was performed using the “lavaan” and “lavaanPlot” R packages. Statistical analysis was performed using IBM SPSS Statistics 22 and R version 4. 2. 3 (R Core Team, 2023). PLS-SEM analysis was performed using SmartPLS: Ringle, C. M., Wende, S., and Becker, J.-M. 2024. “SmartPLS 4.” Bönningstedt: SmartPLS, https://www.smartpls.com.

## 3 Results

### 3.1 Abundance of different functional groups of microorganisms in the rhizosphere

Multivariate analysis of variance (MANOVA) was conducted to examine the effect of season, location, rhizobial treatment, or their interaction on the overall abundance of microorganisms in rhizosphere soil (total number of microorganisms, fungi, oligonitrophiles, and actinomycetes, and MPN of ammonifiers and *Azotobacter*). The results (Wilks’s lambda test) showed that all three factors, namely, season, location, and treatment separately, as well as in interaction (season × location, season × treatment, location × treatment, season × location × treatment), had significant effect on the overall tested microbial groups (*p* < 0.001) (**Supplementary Table 1**). Effect size analysis showed that among the main effects, location (*η*^2^ = 0.959) had the strongest effect, followed by season (*η*^2^ = 0.906) and treatment (*η*^2^ = 0.560).

**Figure 3:**
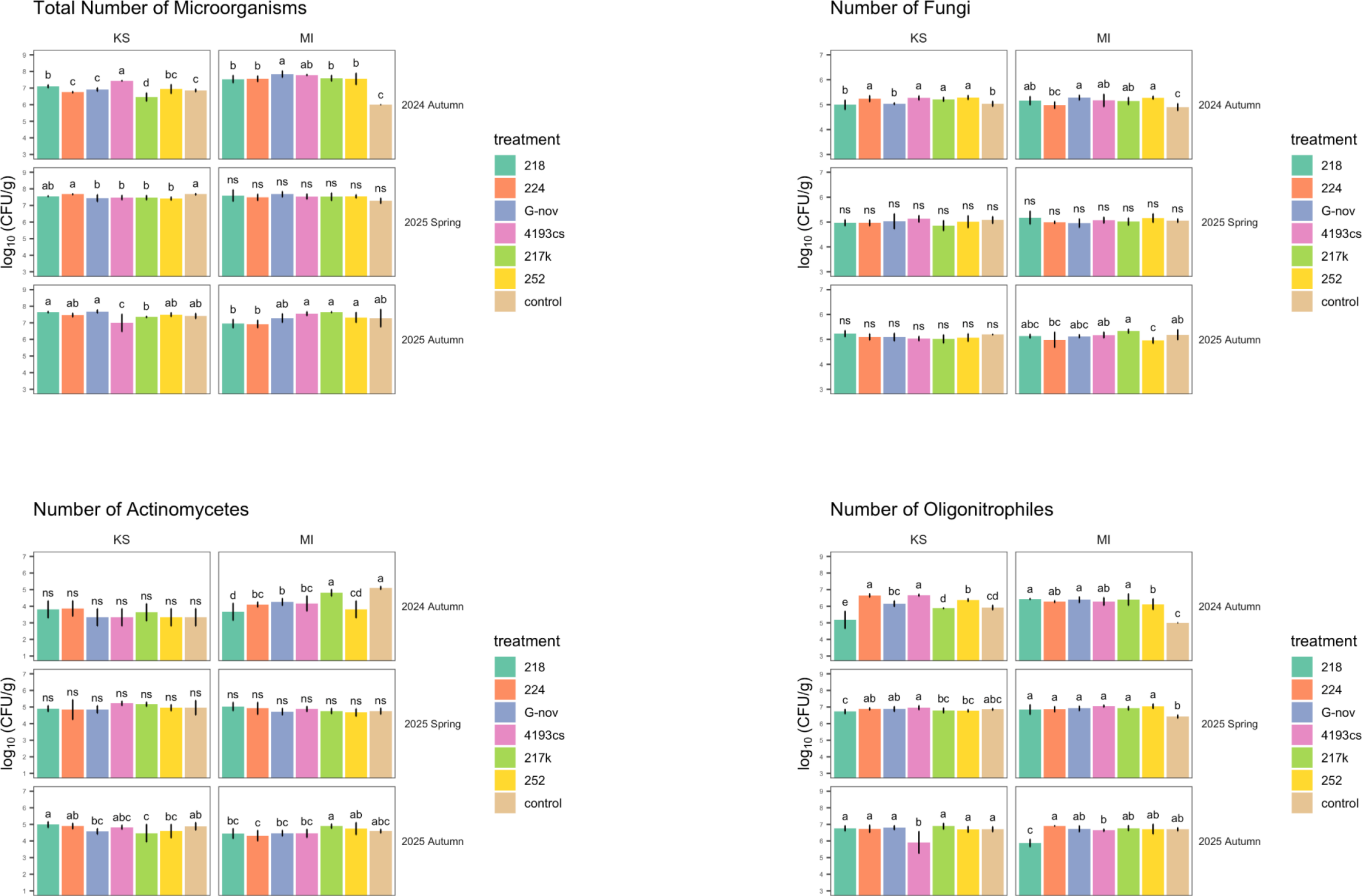
Number of different groups of microorganisms in different seasons in two experimental fields when different inoculants were applied. Different letters indicate statistically significant differences among the different inoculations within the same field and season according to Duncan’s multiple range test (*p* < 0.05).

The numbers of microorganisms in the alfalfa rhizosphere soils belonging to different groups as well as comparison between different treatments within the same field and the same season are given in **Figure 3**. The effect of the factors on each of the microbial groups tested (total number of microorganisms, fungi, oligonitrophiles, and actinomycetes, MPN of *Azotobacter* and ammonifiers, as well as soil respiration rate) is given in **Table 3**.

**Table 3:** Effect of different factors on the number of microbial groups in the rhizosphere of alfalfa plants.

	Log10 (Total number of microorganisms)	Log10 (No. of fungi)	Log10 (No. of oligonitrophiles)	Log10 (MPN of Azotobacter)	Log10 (MPN of ammonifiers)	Log10 (No. of actinomycetes)	Soil respiration rate
Season	***	***	***	***	*	***	***
Location	***	ns	ns	***	***	**	ns
Treatment	***	ns	***	***	**	**	***
Season × location	***	ns	ns	***	***	***	***
Season × treatment	***	***	***	***	***	**	***
Location × treatment	***	*	***	***	***	***	ns
Season × location × treatment	***	**	***	***	***	***	***

ns, no statistical significance; * significant at <0.05; ** significant at <0.01; *** significant at <0.001.

The total number of microorganisms (TNM) was significantly influenced by all tested factors and their interactions (**Table 3**). Considering both locations and all seasons, according to Duncan’s multiple range test, all treatments had higher TNM (from log_10_ CFU/g = 7.31 to log_10_ CFU/g = 7.47) compared to the control (mean value log_10_ CFU/g = 7.17). Among the seasons, the TNM was higher in spring 2025 (mean value log_10_ CFU/g = 7.53), compared to the autumn of the second (mean value log_10_ CFU/g = 7.36) and the first years (mean value log_10_ CFU/g = 7.17).

When observing the results within one field and one season (**Figure 3**), in autumn 2024, compared to the control, TNM was the highest in the soil sample collected from the rhizosphere of alfalfa inoculated with 4193cs, followed by 218 in the KS location (higher Ni concentration), and in the rhizosphere soil of all inoculated treatments in MI. In spring 2025 (**Figure 3**), in the KS location, 218 and 224 did not differ statistically from the control, while other groups had lower TNM compared to the control. In the MI location in the same season, no significant differences among the experimental groups were detected. In autumn 2025 (**Figure 3**), in KS, significantly lower TNM was detected in the group inoculated with 4193cs. In the same season, in the MI location, there were no statistically significant differences in TNM in any of the inoculated treatments compared to the control (**Figure 3**).

The number of actinomycetes was significantly influenced by all tested factors and their interactions (**Table 3**). Considering both locations and all seasons, according to Duncan’s multiple range test, none of the inoculated treatments had significantly higher number of actinomycetes compared to the control. Among the seasons, the number of actinomycetes was also the highest in spring 2025 (mean value log_10_ CFU/g = 4.90) compared to the autumn of the first (mean value log_10_ CFU/g =3.90) and second years (mean value log_10_ CFU/g = 4.66).

In autumn 2024 (**Figure 3**), all inoculated groups, except 217k, had significantly lower number of actinomycetes compared to the uninoculated control in MI, while there were no significant differences in the KS location. In spring 2025, there were no statistically significant differences in the number of actinomycetes among the treatments in both locations. In autumn 2025 as well, one of the inoculated groups (217k) in the KS location had a statistically lower number of actinomycetes compared to the control, while none of the inoculated groups differed statistically from the control in MI (**Figure 3**).

The number of fungi was significantly influenced by season and its interaction with location or location and treatment. Treatment and location separately had no effect on the number of fungi, but their interaction did (**Table 3**). Across all the locations and treatments, according to Duncan’s multiple range test, the highest number of fungi was in the autumn of the first year (mean value log_10_ CFU/g = 5.14), similar to the autumn of the second year (mean value log_10_ CFU/g = 5.12), and a significantly lower number of fungi was in the spring of the first year (mean value log_10_ CFU/g = 5.03).

Within one field and one season (**Figure 3**), compared to the uninoculated control, the number of fungi was higher in the rhizosphere of alfalfa of all inoculated treatments, except 218 and G-nov, in autumn 2024 in KS, and in all inoculated treatments, except 224, in MI in the same season (**Figure 3**). In spring 2025, there were no statistically significant differences among the treatments in both locations, as well as in autumn 2025 in KS. In autumn 2025 in MI, only 252 had a statistically lower number of fungi compared to the control (**Figure 3**).

The number of oligonitrophiles was significantly affected by all factors and their interactions, except for location and the interaction between location and season (**Table 3**). According to Duncan’s multiple range test, across all treatments and locations, the highest number of oligonitrophiles was in the spring (mean value log_10_ CFU/g = 6.85), followed by the autumn of the second (mean value log_10_ CFU/g = 6.64) and then the first year (mean value log_10_ CFU/g = 6.13). All inoculated treatments, except 218, had a higher number of oligonitrophiles (from log_10_ CFU/g = 6.59 to log_10_ CFU/g = 7.72) compared to the uninoculated control (mean value log_10_ CFU/g = 6.27).

In autumn 2024, a higher number of oligonitrophiles were detected in the rhizosphere of plants inoculated with 224, 4193cs, and 252 in KS and in all the inoculated treatments in MI, compared to the control. In spring 2025, all inoculated groups had a significantly greater number of oligonitrophiles compared to the control in MI, while there was no difference between the control and other treatments in KS. In autumn 2025, a significantly lower number of oligonitrophiles compared to the control were observed in the rhizosphere of plants treated with 4193cs in KS and with 218 in MI, while the other groups did not differ significantly from the control (**Figure 3**).

The MPN of *Azotobacter* was significantly influenced by all factors and their interactions. The highest MPN of *Azotobacter* was in the autumn of the second year (mean value log_10_ MPN/g = 2.23), followed by the spring of the second year and the autumn of the first year, which had similar numbers (mean value log_10_ MPN/g = 2.1 and 2.07, respectively). Compared to the control (log_10_ MPN/g = 2.04), the treatments 217k, 4193cs, G-nov, and 224 had greater MPN (from log_10_ MPN/g = 2.09 to log_10_ MPN/g = 2.33) of *Azotobacter* across all seasons and locations.

**Figure 4:**
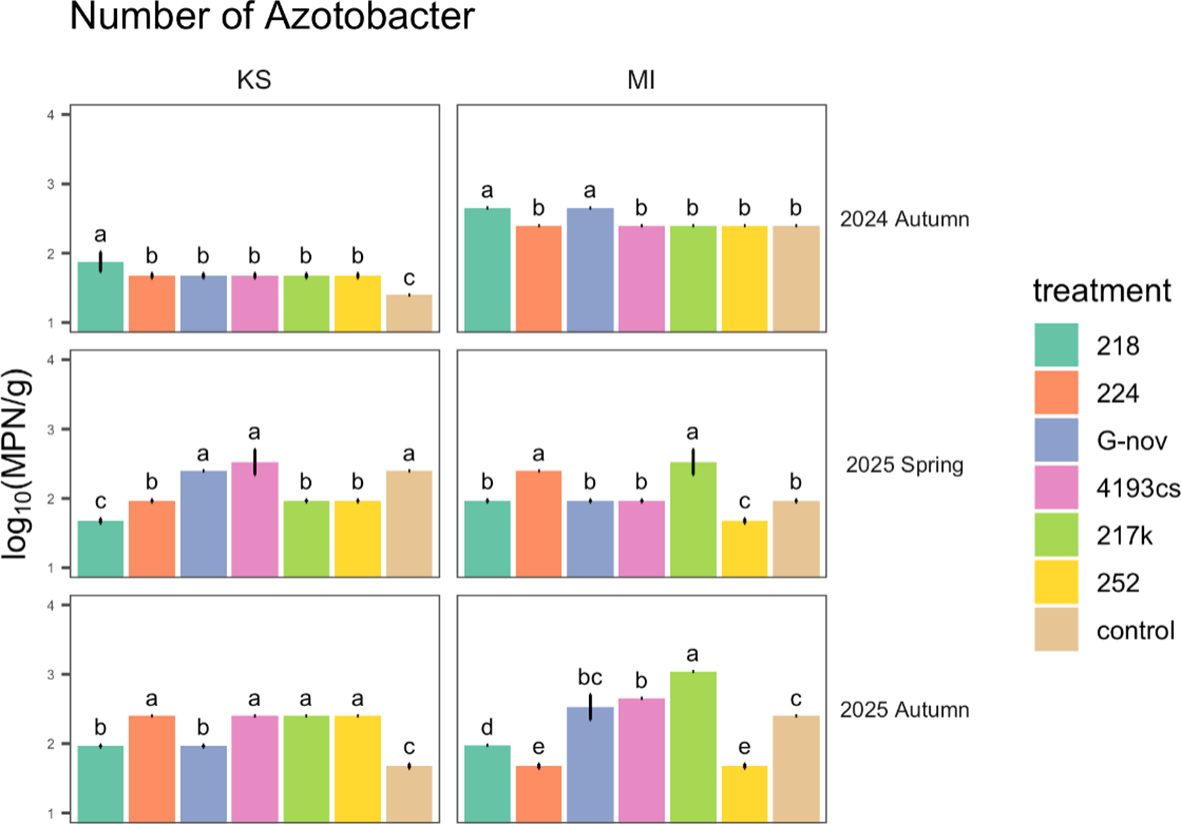
Most probable number of *Azotobacter* different seasons in two experimental fields when different inoculants were applied. Different letters indicate the statistically significant differences among the different inoculations within the same field and season according to Duncan’s multiple range test (*p* <0.05).

Within one field and one season (**Figure 4**), in autumn 2024, all inoculated treatments in KS and in the groups treated with 218 and G-nov in MI had a greater MPN of *Azotobacter* compared to the uninoculated control. In spring 2025, two treatments in KS (G-nov and 4193cs) had similar MPN, and others had lower MPN of *Azotobacter*. In MI, 224 and 217k had higher MPN, and 252 had lower MPN of *Azotobacter* compared to the control. In autumn 2025, all inoculated treatments in KS, as well as 4193cs and 217k treatments in MI, had higher MPN compared to the control, while treatments 218, 224, and 252 in MI had lower MPN of *Azotobacter* compared to the control (**Figure 4**).

The MPN of ammonifiers was significantly influenced by all factors and their interactions. According to Duncan’s multiple range test, across all treatments and locations, the significantly highest number of ammonifiers was in the spring (mean value log_10_ MPN/g = 6.71) compared to the autumn of the second (mean value log_10_ MPN/g = 6.48) and first years (mean value log_10_ MPN/g = 6.32), which did not differ statistically from each other. None of the treatments had significantly greater MPN of ammonifiers compared to the control.

**Figure 5:**
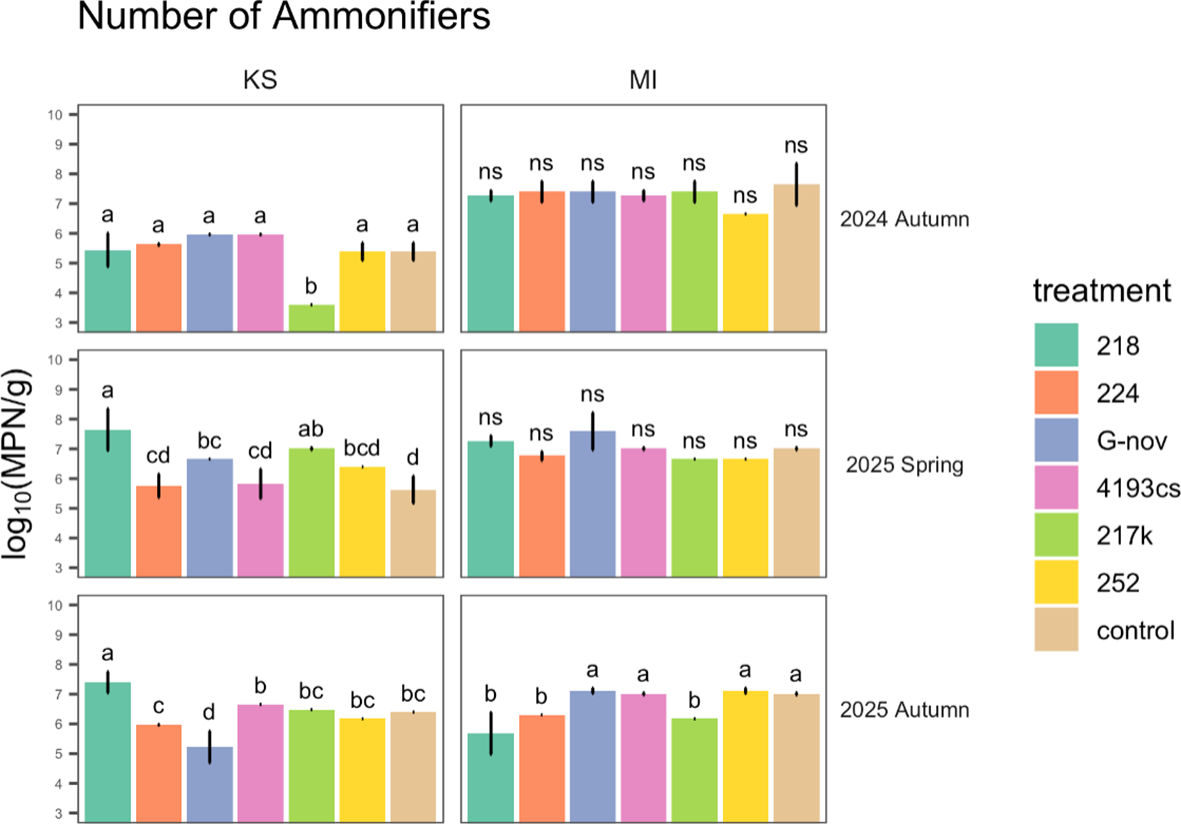
Most probable number of ammonifiers different seasons in two experimental fields when different inoculants were applied. Different letters indicate the statistically significant differences among the different inoculations within the same field and season according to Duncan’s multiple range test (*p* <0.05).

In autumn 2024 (**Figure 5**), the 217k treatment in KS had lower MPN of ammonifiers compared to the control, while in MI, there were no significant differences among the treatments. In spring 2025, the 218, G-nov, and 217k treatments in KS had greater MPN of ammonifiers compared to the control, while in MI, there were no statistically significant differences among the treatments. In autumn 2025, the group treated with 218 in KS had greater MPN, while the group treated with G-nov had lower MPN compared to the control. In the same season, in MI, 218, 224, and 217k had lower MPN compared to the control.

Significant positive correlations between the tested microbial groups were detected, where the most significant correlations were between the TNM in the location MI in two seasons in 2024 and spring 2025 (*r* = 0.906**, *p* < 0.01) and the TNM and the number of oligonitrophiles in season 2024 (*r* = 0.967**, *p* < 0.01) and in spring 2025 (*r* = 0.775*, *p* < 0.05) as well as the TNM and the number of oligonitrophiles in the location KS in spring 2025 (*r* = 0.822**, *p* < 0.05).

### 3.2 Basal soil respiration rate

**Figure 6:**
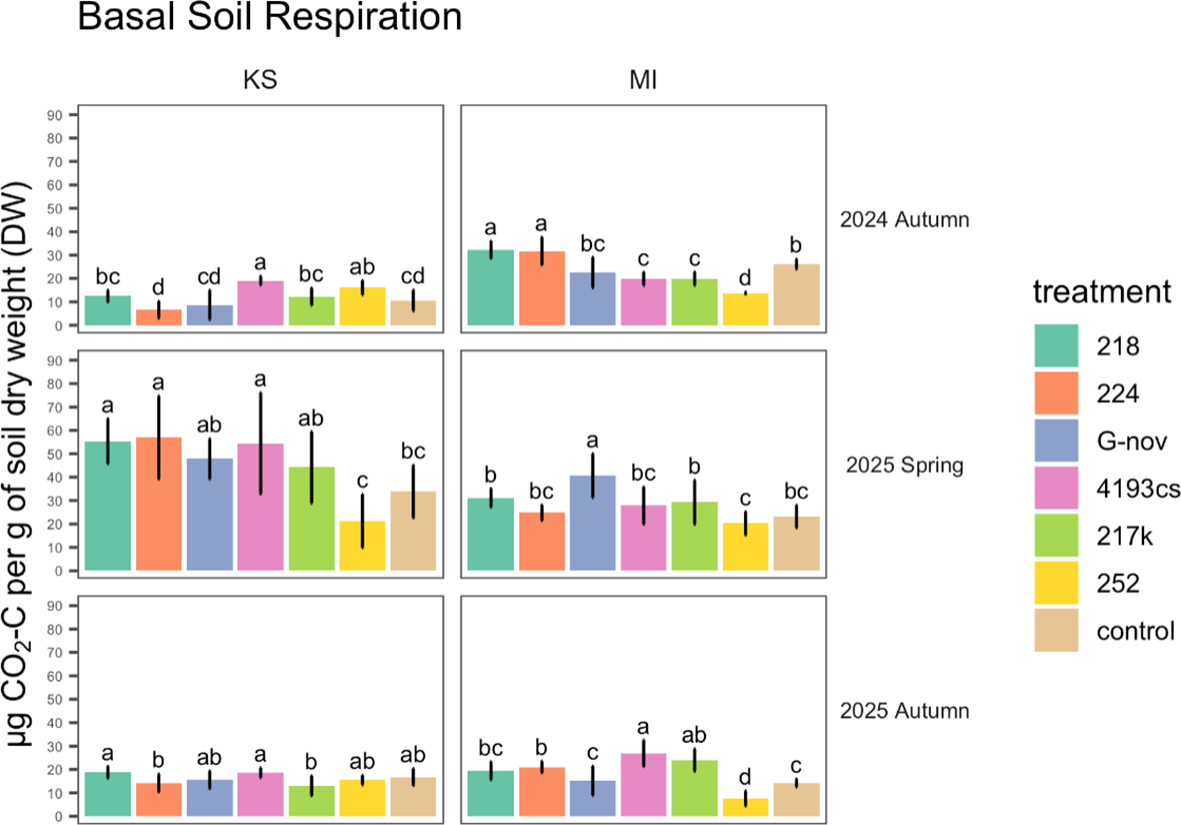
Soil respiration rates in different seasons in two experimental fields when different inoculants were applied. Different letters indicate the statistically significant differences among the different inoculations within the same field and season according to Duncan’s multiple range test (*p* <0.05).

The basal soil respiration (BSR) was not significantly influenced by location or the interaction between location and treatment, but depended on treatment and season, as well as all the other interactions between these factors (**Table 3**). Considering both locations and all seasons, the BSR was the highest in the rhizosphere of the 218 treatment (28.27 μg CO_2_-C/g) and the lowest in the 252 treatment (15.75 μg CO_2_-C/g), followed by the control (20.80 μg CO_2_-C/g). Among the seasons, the BSR was significantly higher in the spring (36.52 μg CO_2_-C/g), while it had approximately the same average value in the autumn of both years (18.03 in the first year and 17.21 μg CO_2_-C/g in the second year). Basal soil respiration rate was significantly lower in the experimental groups inoculated with 4193cs, 217k, and 252 in MI, while inoculation with 4193cs and 252 in KS or with 218 and 224 in MI resulted in a statistically greater respiration rate compared to the control, in autumn 2024 (**Figure 6**). In spring 2025, the experimental groups that were inoculated with 218, 224, and 4193cs in KS and G-nov in MI had a greater respiration rate compared to the uninoculated control (**Figure 6**). In autumn 2025, inoculation with 224, 4193cs, and 217k resulted in greater BSR, while inoculation with 252 resulted in lower BSR compared to the control in MI. In KS, in autumn 2025, all inoculated groups had similar BSR compared to the control (**Figure 6**).

### 3.3 Growth of alfalfa

It was observed that alfalfa grew well in both locations with and without inoculation, with no visible symptoms of toxicity. In all the inoculated treatments, approximately the same number of nodules was noticed, with similar positions across the root (**Supplementary Figure 1**). The inoculation of alfalfa in particular treatments increased alfalfa yield in both years as well as locations compared to the control uninoculated plants (**Table 4**). The yield depended on the treatments (*p* < 0.05) (first year) or treatment and location (*p* < 0.05) (second year). In both years and locations, the best treatments were 4143cs, G-nov, and 252 (**Table 4**). The average increase in treatments (for both years and locations) was up to 38% increase compared to the control plants (**Supplementary Material 3**). The yield significantly correlated with the total number of microorganisms in the location MI in the first year (*r* = 0.852, *p* < 0.05).

**Table 4:** Shoot dry weight of alfalfa.

Treatment		Shoot dry weight (g/m2)	
		1st year autumn	2nd year autumn
Location MI (lower Ni)	218	52.51 ± 17.70 ab	215.60 ± 19.88 c
	224	61.54 ± 23.07 ab	245.16 ± 17.79 ab
	G-nov	73.64 ± 16.21 a	290.04 ± 63.72 a
	4193cs	78.01 ± 20.60 a	307.51 ± 88.74 a
	217k	59.45 ± 7.14 ab	260.22 ± 28.92 ab
	252	56.36 ± 20.08 ab	213.68 ± 29.85 c
	Control	36.35 ± 3.86 b	192.30 ± 28.44 c
Location KS (higher Ni)	218	113.17 ± 20.13 abc	123.08 ± 10.72 c
	224	130.14 ± 12.93 abc	120.35 ± 76.43 c
	G-nov	133.10 ± 10.16 abc	192.18 ± 27.53 ab
	4193cs	114.11 ± 21.26 bc	197.28 ± 57.37 ab
	217k	137.46 ± 1.80 ab	165.00 ± 14.94 abc
	252	147.73 ± 14.30 a	218.79 ± 40.84 a
	Control	129.78 ± 12.55 c	148.30 ± 29.91 bc
Source of variation		*p*-value	
Treatment		0.000	0.000
Location		0.056	0.000
Location × treatment		0.011	0.080

a–c: Values in a column within the same location marked with the same letters are not statistically different according to Duncan’s multiple range test (*p* < 0.05).

### 3.4 Soil chemical properties

Soil chemical characteristics in the autumn of the second year of cultivation showed that pH was influenced by treatment and location (*p* < 0.05). In the location KS, pH did not vary among the treatments, while in the MI location, it was increased in some treatments (**Table 5**). There were no differences among the treatments in N%, only among locations (*p* < 0.05), while SOM did not differ among the treatments and locations. The values of available elements in the soil were influenced by location and for some elements also by treatments (*p* < 0.05) (Cu and Mn) (**Table 6**). There were negative correlations between pH and TNM (*r* = −0.784, *p* < 0.05) as well as pH and fungi (*r* = −0.865, *p* < 0.01) in the MI location.

**Table 5:** Chemical properties of soils at the end of the second experimental year (2025).

Treatment		pH (H_2_O)	N%	SOM %
Location MI (lower Ni)	218	7.37 ± 0.02 bc	0.14 ± 0.01 a	2.46 ± 0.11 a
	224	7.65 ± 0.03 a	0.15 ± 0.01 a	2.60 ± 0.11 a
	G-nov	7.17 ± 0.14 cd	0.16 ± 0.01 a	2.63 ± 0.18 a
	4193cs	7.12 ± 0.01 d	0.17 ± 0.02 a	2.85 ± 0.21 a
	217k	7.08 ± 0.02 d	0.16 ± 0.01 a	2.65 ± 0.21 a
	252	7.48 ± 0.20 ab	0.16 ± 0.01 a	2.62 ± 0.31 a
	Control	7.21 ± 0.03 cd	0.16 ± 0.01a	2.64 ± 0.11 a
Location KS (higher Ni)	218	7.66 ± 0.01 a	0.20 ± 0.03 a	2.93 ± 0.20 a
	224	7.50 ± 0.09 a	0.18 ± 0.01 a	2.64 ± 0.25 a
	G-nov	7.59 ± 0.05 a	0.20 ± 0.00 a	2.69 ± 0.24 a
	4193cs	7.61 ± 0.04 a	0.20 ± 0.01 a	2.80 ± 0.18 a
	217k	7.65 ± 0.16 a	0.19 ± 0.00 a	2.83 ± 0.00 a
	252	7.63 ± 0.11 a	0.17 ± 0.01 a	2.46 ± 0.16 a
	Control	7.59 ± 0.08 a	0.19 ± 0.00 a	2.64 ± 0.02 a
Source of variation		*p*-value		
Treatment		0.016	0.380	0.469
Location		0.000	0.000	0.275
Location × treatment		0.002	0.312	0.346

a–c: Values in a column within the same location marked with the same letters are not statistically different according to Duncan’s multiple range test (*p* < 0.05).

**Table 6:** Concentrations of trace elements in two experimental fields at the end of the second experimental year (2025).

Treatment		Trace elements (available concentrations) (mg/kg)									
		As	Cd	Co	Cr	Cu	Fe	Mn	Ni	Pb	Zn
Location MI (lower Ni)	218	0.065 ± 0.014	0.035 ± 0.002	0.095 ± 0.035	0.007 ± 0.001	1.650 ± 0.018	29.53 ± 3.37	16.01 ± 2.80	1.426 ± 0.098	1.262 ± 0.090	0.819 ± 0.049
	224	0.067 ± 0.025	0.035 ± 0.004	0.105 ± 0.060	0.007 ± 0.001	1.532 ± 0.176	28.07 ± 3.78	17.39 ± 1.952	1.650 ± 0.284	1.132 ± 0.056	0.850 ± 0.227
	G-nov	0.065 ± 0.007	0.036 ± 0.000	0.107 ± 0.008	0.007 ± 0.000	1.667 ± 0.097	31.17 ± 2.83	17.65 ± 0.877	1.592 ± 0.149	1.332 ± 0.199	0.972 ± 0.031
	4193cs	0.064 ± 0.007	0.037 ± 0.002	0.110 ± 0.030	0.008 ± 0.001	1.635 ± 0.012	34.33 ± 5.36	18.74 ± 3.267	1.720 ± 0.387	1.518 ± 0.113	1.060 ± 0.027
	217k	0.063 ± 0.008	0.038 ± 0.000	0.097 ± 0.014	0.007 ± 0.001	1.756 ± 0.019	33.08 ± 0.18	16.78 ± 1.35	1.628 ± 0.129	1.368 ± 0.101	1.145 ± 0.113
	252	0.059 ± 0.008	0.035 ± 0.001	0.075 ± 0.016	0.006 ± 0.000	1.545 ± 0.077	27.71 ± 1.40	13.86 ± 1.71	1.207 ± 0.158	1.346 ± 0.049	0.920 ± 0.079
	Control	0.059 ± 0.002	0.036 ± 0.003	0.083 ± 0.011	0.007 ± 0.000	1.596± 0.050	30.83 ± 3.69	15.35 ± 1.33	1.447 ± 0.215	1.387 ± 0.030	1.133 ± 0.344
Location KS (higher Ni)	218	0.041 ± 0.000	0.116 ± 0.003	0.117 ± 0.002a	0.009 ± 0.000b	1.797 ± 0.006c	21.27 ± 0.17	15.17 ± 0.39b	4.331 ± 0.153	12.270 ± 0.311	2.328 ± 0.066
	224	0.046 ± 0.001	0.120 ± 0.004	0.134 ± 0.013a	0.009 ± 0.000a	2.034 ± 0.282bc	21.08 ± 0.21	17.03 ± 0.83a	4.963 ± 0.337	12.735 ± 0.021	2.567 ± 0.196
	G-nov	0.043 ± 0.003	0.119 ± 0.005	0.119 ± 0.010a	0.009 ± 0.000ab	2.035 ± 0.194bc	20.00 ± 0.99	15.44 ± 1.31ab	4.723 ± 0.290	12.205 ± 0.318	2.581 ± 0.184
	4193cs	0.043 ± 0.001	0.114 ± 0.003	0.114 ± 0.001a	0.009 ± 0.000ab	2.251 ± 0.264ab	20.61 ± 0.21	15.37 ± 0.045ab	4.458 ± 0.110	12.115 ± 0.361	2.424 ± 0.126
	217k	0.041 ± 0.003	0.115 ± 0.004	0.125 ± 0.004a	0.009 ± 0.000ab	2.517 ± 0.152a	21.52 ± 2.08	16.11 ± 0.29ab	4.324 ± 0.276	12.515 ± 0.078	2.448 ± 0.053
	252	0.042 ± 0.001	0.114 ± 0.003	0.116 ± 0.012a	0.009 ± 0.000ab	1.822 ± 0.028bc	20.26 ± 1.27	14.91 ± 0.87b	4.526 ± 0.257	12.080 ± 0.523	2.450 ± 0.042
	Control	0.042 ± 0.001	0.112 ± 0.005	0.063 ± 0.001b	0.008 ± 0.000c	1.746 ± 0.037c	18.92 ± 1.43	11.35 ± 0.15c	4.048 ± 0.161	11.455 ± 0.035	2.544 ± 0.196
Source of variation		*p*-value									
Treatment		0.939	0.634	0.130	0.075	0.003	0.392	0.024	0.054	0.075	0.323
Location		0.000	0.000	0.065	0.000	0.000	0.000	0.023	0.000	0.000	0.000
Location × treatment		0.996	0.382	0.578	0.487	0.045	0.429	0.299	0.204	0.008	0.531

a–c: Values in a column within the same location marked with the same letters are not statistically different according to Duncan’s multiple range test (*p* < 0.05).

### 3.5 Potential influence of factors on soil microorganisms and alfalfa yield

Stepwise multiple regression analysis was used to investigate and model the relationships between the alfalfa yield and the chemical and microbiological properties of soil samples, aiming to identify microbiology groups with the highest influence. The analysis showed that the total number of microorganisms was a significant predictor of shoot dry weight (location KS in 2024; adjusted *R*-squared, *R*^2^ = 0.671*, *p* < 0.05). The number of fungi and the MPN of ammonifiers were significant predictors of pH (location MI in autumn 2025; *R*^2^ = 0.873*, *p* < 0.05), and the total number of microorganisms was a significant predictor of %N (location MI in autumn 2025; *R*^2^ = 0.573*, *p* < 0.05).

**Figure 7:**
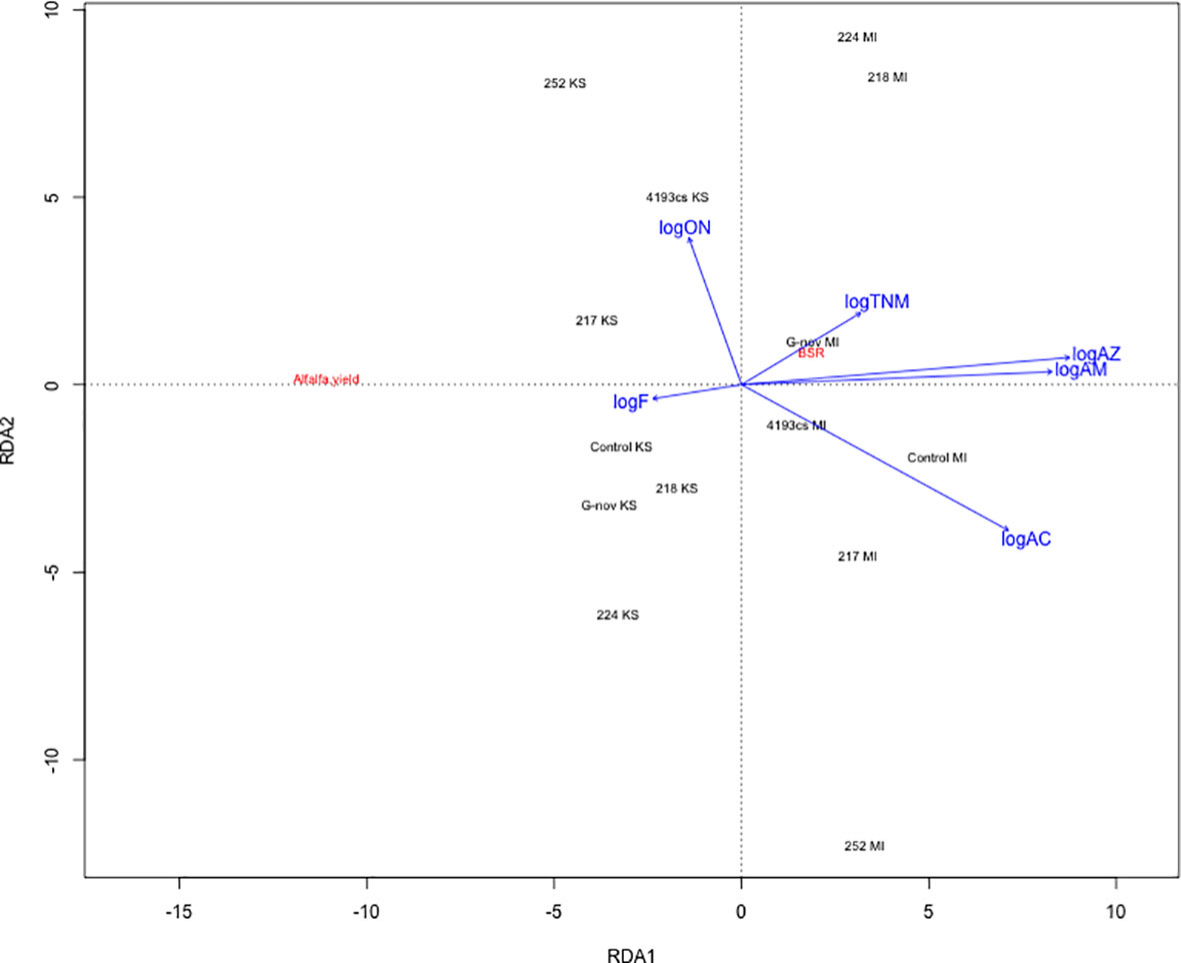
Redundancy analysis (RDA) ordination plot showing variation in (yield of alfalfa and basal soil respiration in 2024) explained by (abundance of different microbial groups). Axes RDA1 and RDA2 represent the first two constrained components, accounting for 89.51% and 0.5289% of the variance, respectively, suggesting that most variation is explained by one main gradient.

The RDA model for the season 2024 was significant (*p* = 0.004), and the variation in response variables (basal soil respiration rate and yield in 2024) can be explained by explanatory variables (number of different microbial groups) (constrained variance = 1377, larger than the unconstrained variance = 152.3) (**Figure 7**). The eigenvalues for RDA1, RDA2, PC1, and PC2 were 1368.9086, 8.089247, 133.79707, and 18.51443, respectively, and the proportion of variance explained by these axes was as follows: 89.51%, 0.53%, 8.75%, and 1.21%. Among the explanatory variables, the total number of microorganisms (*F* = 7.6118, *p* = 0.025) and the MPN of *Azotobacter* (*F* = 51.7234, *p* = 0.001) had significant effects. RDA1 was strongly associated with the number of *Azotobacter*, while TNM was oriented between the two axes, indicating that it was correlated with both gradients (**Figure 7**). The RDA model for the season autumn 2025 was not significant (*p* = 0.290).

**Figure 8:**
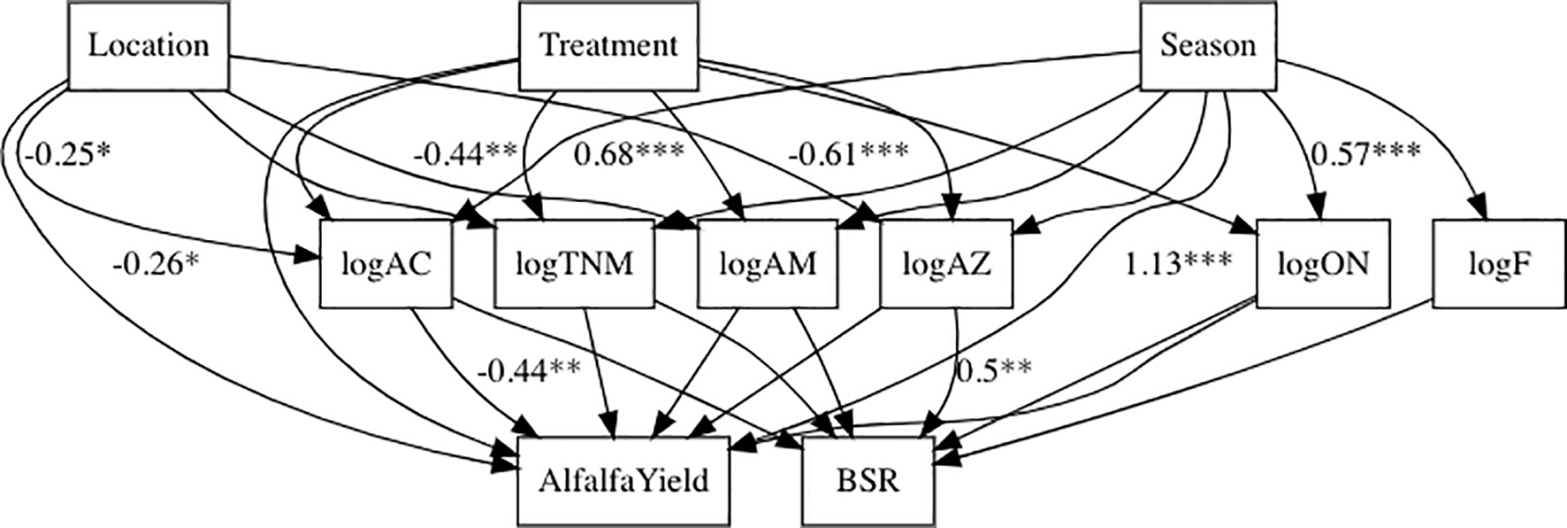
Structural equation model (SEM) shows the hypothesized model of relationships among alfalfa yield, microbiological properties (logTNM, logF, logAZ, logAM, logON, logAC), basal soil respiration (BSR), location, inoculation and season. The model showed a good fit to the data, with χ^2^=20.477, *df*=14, p=0.116, CFI=0.953. Numbers on arrows represent significant path coefficients. *R*^2^ values represent the proportion of the variance explained for each variable (logTNM = 0.151; logAC = 0.534; logAZ = 0.395; logAM = 0.270; logF = 0.008; logON = 0.323; Alfalfa yield = 0.799; BSR = 0.409).

Structural equation modeling (SEM) was performed to investigate the relationships among variables including location, season, inoculation, numbers of different microorganisms, basal soil respiration rate, and alfalfa yield (**Figure 8**). This model fits our hypothesis (*χ*^2^ = 20.48, *df* = 14, *p* = 0.12, CFI = 0.95). The effect of season on the number of oligonitrophiles (0.57) and alfalfa yield (1.13) was significantly positive. The effect of location on the number of actinomycetes (−0.26), *Azotobacter* (−0.61), ammonifiers (−0.44), and alfalfa yield (−0.25) was significantly negative. Actinomycetes had a significantly negative effect on alfalfa yield (−0.44). *Azotobacter* had a significantly positive effect on basal soil respiration (0.50). The final model explained 15.1% variance for TNM, 53.4% for the number of actinomycetes, 39.5% for *Azotobacter*, 27.0% for the number of ammonifiers, 0.8% for the number of fungi, 32.3% for the number of oligonitrophiles, 79.9% for alfalfa yield, and 40.9% for BSR.

**Figure 9:**
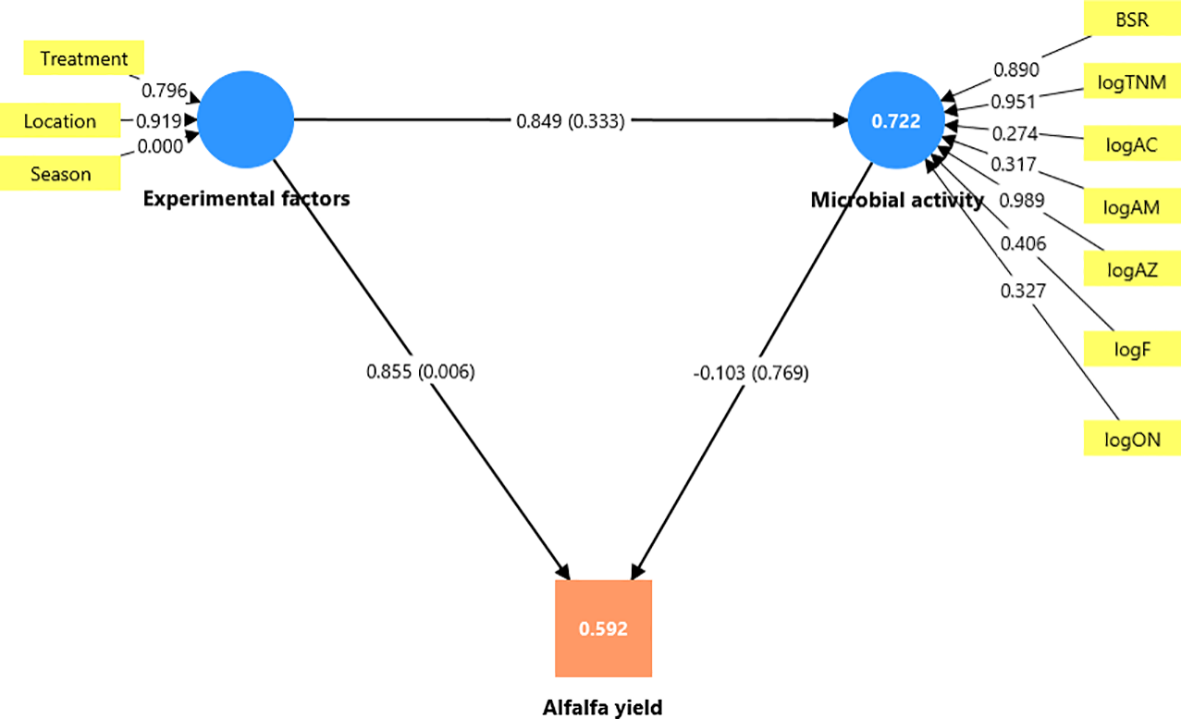
The PLS-SEM model. Numbers on the arrows indicate *β* coefficients and p-values, while numbers inside the alfalfa yield and microbial activity fields indicate *R*^2^ values.

PLS-SEM direct path analysis showed that experimental factors (*b* = 0.855; *p* = 0.006) had a statistically significant effect on alfalfa yield but did not have a significant effect on microbial activity (*b* = 0.849; *p* = 0.333), while microbial activity (*b* = −0.103; *p* = 0.769) did not have a significant effect on alfalfa yield (**Figure 9**).

## 4 Discussion

Potentially toxic elements include highly toxic heavy metals without physiological function (Cr, Pb, Hg, and Cd) and biologically essential heavy metals (Mo, Mn, Fe, Co, Ni, Cu, Zn) that can be highly toxic at high concentrations (Nieder and Benbi, 2023). The negative impact of heavy metals on microorganisms in the soil can be evaluated through measuring different microbial properties, such as microbial activity (microbial or fungal growth, soil basal respiration, and enzymatic activities), microbial community composition and/or diversity [DNA-based analyses, culture-dependent techniques, and phospholipid fatty acid (PLFA) analysis], and microbial biomass/abundance (Campillo-Cora et al., 2025). Although culturable microorganisms represent a small fraction of the microbial community, some of them are sufficiently sensitive to be used as bioindicators in soils exposed to contamination (Gorovtsov et al., 2018).

In this study, we evaluated the effect of two soils containing increasing contents of different metals, mainly Ni, Pb, and Cr, on the functional soil microbial communities. The soils investigated in this research have heavy metal concentrations below the remediation levels and come from natural geogenic sources. These soils generally have good physical and chemical properties, but more research is required regarding plant growth, particularly how these concentrations affect the indigenous microflora or microorganisms introduced by inoculation. Therefore, one of the goals in this study was to evaluate the effect of rhizobial preinoculation on microbial groups in response to elevated heavy metal contents. Soil samples were taken from the rhizosphere of alfalfa plants, a very active and dynamic zone where plant–microorganisms interactions take place.

It was found that the total number of microorganisms and some groups such as actinomycetes, *Azotobacter*, and ammonifiers were under the influence of location, being somewhat higher in the treatments in soil with lower Ni. However, the other microbial groups as well as basal soil respiration were not under the influence of location. This could be the result of slightly increased Ni and other heavy metal concentrations in our tested fields. However, addressing these results is complex, as a multitude of interacting factors shape the microbial communities in the soil and modulate the effects of metals on soil microorganisms. The toxicity of heavy metals is affected by soil properties including pH, organic matter content, and texture as these factors determine the availability of heavy metals (Campillo-Cora et al., 2025). Previously, the synergistic effects of heavy metals and soil physicochemical properties on the bacterial communities were confirmed, and the results revealed that microbial structure was strongly shaped by soil pH, total Cd, and available As (Hu et al., 2021). A more systematic overview, performed through a meta-analysis using data collected from a large number of published studies addressing the effect of heavy metals on soil microorganisms (Li et al., 2025), indicated negative effects on bacterial and fungal abundance, microbial biomass carbon and nitrogen, and the activity of the enzymes arylsulfatase and dehydrogenase. This meta-analysis further indicated that there were more pronounced negative effects of heavy metals on microbial abundance than on microbial diversity.

On the other hand, trace elements represent a strong selective pressure that favours tolerant microorganisms possessing mechanisms of efflux and detoxification of trace elements, thereby conferring resistance (Nikolova et al., 2025). A study by Li et al. (2017) showed that long-term exposure to heavy metals caused changes in microbial community composition more than in its diversity and that changes in interactions among microbes might be the way microbes adapt to heavy metal contamination. It is widely known that bacteria exchange mobile genetic elements through horizontal gene transfer, enabling them to acquire resistance to heavy metals and antibiotics, as these genes are usually located on mobile genetic elements. A previous study confirmed that contamination with Cu and Zn along a sewage pipe resulted in higher abundance of heavy metal resistance genes, obtained through horizontal gene transfer, among the bacteria in regions of the sewage pipe where higher concentrations of Cu and Zn were detected (Wu et al., 2025). In our previous study (Pešić et al., 2025), we confirmed that our rhizobial strains had a certain level of nickel tolerance *in vitro*, and the concentrations we tested were much higher compared to those present in both experimental fields; therefore, we assumed that Ni present in the field could not interfere with the survival of our inoculants.

In our research, all tested groups of microorganisms (except fungi) were under the influence of treatments, which overall had positive or no influence on their values. Previously, inoculation modified the number of microaerophilic nitrogen-fixing microorganisms at the reproductive stage of maize, while it did not affect the amount of cellulolytic and nitrifying microorganisms (Di Salvo et al., 2018). Inoculation with *E. meliloti* had positive effects on microbial properties (increased soil enzyme activities of β-glucosidase and alkaline phosphatase and microbial biomass nitrogen) in soils under Cu stress (Duan et al., 2022). Inoculation with two bacterial strains influenced the soil bacterial and fungal community structure in the rhizosphere and rhizoplane of rice 65 days after inoculation, with more significant changes observed in the rhizoplane (Huang et al., 2024). In our study, the rhizobial treatment alone also had a significant influence on the soil respiration rate, which indicates that rhizobia affected the activity of microorganisms in the rhizosphere. In another study, soil respiration rate and microbial biomass N in the rhizosphere zone were affected by the interaction between the inoculation with the rhizobacteria *Azospirillum brasilense* and *Pseudomonas fluorescens* and the dose of N fertilizer applied (Zhang et al., 2021). In a study by Wang et al. (2007), the results showed that microbial biomass C, soil phosphatase activity, and community diversity were negatively affected by the extractable and total contents of Cu and Zn. The soil used for testing was sampled at increasing distances from the Cu–Zn smelter and, consequently, contained decreasing contents of these elements as the distance increased.

Season alone significantly affected all evaluated microbiological soil properties, suggesting the importance of weather conditions as the key factor determining the soil microbial fluctuations. In addition, the interactions of treatment, season, and location influenced almost all evaluated microbiological soil properties. It was found that seasonal changes were the dominant factor affecting soil microbial biomass and community structure, frequently outweighing the effects of long-term fertilization, tillage, or crop rotation (Kovács et al., 2025).

It is well known that rhizobia are beneficial bacteria that enable leguminous plants to use atmospheric nitrogen (N_2_) (Lindström and Mousavi, 2019), but besides nitrogen availability to the plants, rhizobia possess other traits, including phosphate solubilization, production of phytohormones (auxins, cytokinins, gibberellins, abscisic acid) or compounds that regulate the level of plant hormones [aminocyclopropane-1-carboxylate (ACC) deaminase], siderophore production, and biocontrol and protection from plant pathogens (Fahde et al., 2023) that help plants grow, overcome biotic and abiotic stress, and thrive in a challenging environment. The strains used here, besides possessing N-fixation ability, all have multiple PGP characteristics, such as IAA production, phosphate solubilization, and ACC deaminase production (**Supplementary Table 2**), which might be involved in plant growth promotion as well as heavy metal stress elevation. Moreover, all the strains exhibited a certain percentage of nickel adsorption potential *in vitro*, which can also be responsible for their PGP effect under soil contamination.

The effect of inoculation treatments on soil chemical properties was not high and was noticeable through changes in pH and slight variations in Cu and Mn contents, which is in agreement with previous findings that changes in chemical soil properties are more strongly influenced by other measures than by bacterial inoculation (Ursu et al., 2025).

**Figure 10:**
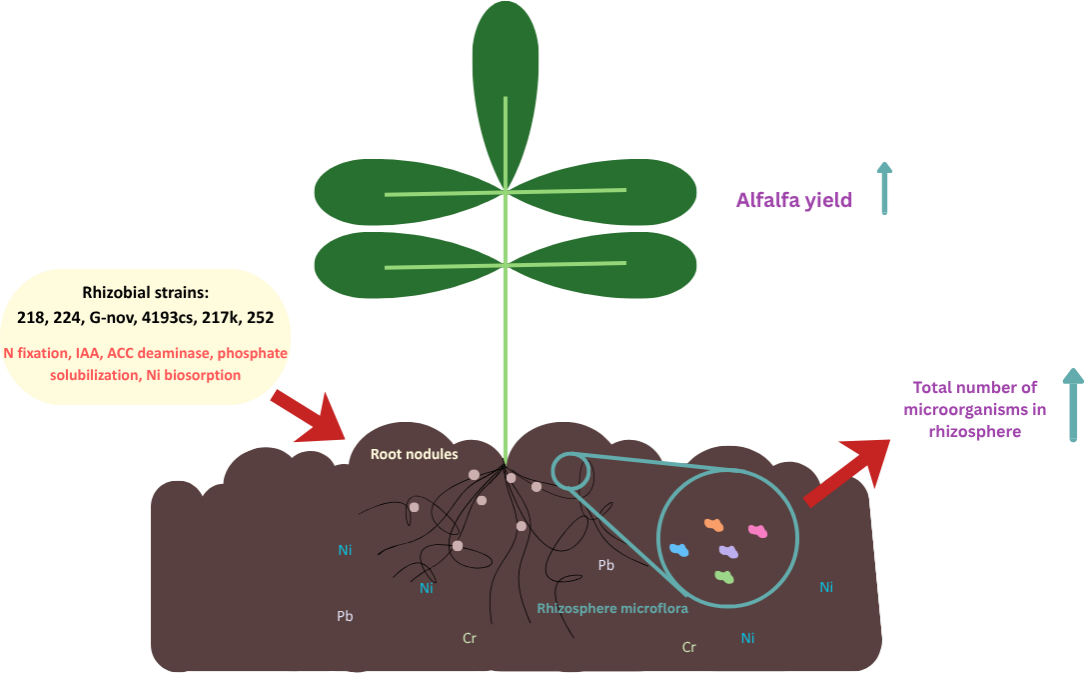
Effect of rhizobial bacteria inoculation.

Previous studies under controlled conditions and artificial contamination indicated that different plant species in association with rhizobial strains reached higher values of different plant growth parameters in soils amended with Ni or other metals (Edulamudi et al., 2022; Elbagory et al., 2022; Duan et al., 2022; Pacheco-Insausti et al., 2023). In our study, specific strains of *E. meliloti* were able to promote growth in alfalfa plants in soils with elevated concentrations of Ni compared to non-inoculated control under field conditions. The increase in shoot dry weight of alfalfa is mainly the result of the effectiveness of nitrogen fixation of the symbiosis between rhizobia and alfalfa, with SDW being the most valuable indicator of symbiotic effectiveness in legume–rhizobia relationships (Han et al., 2024). In the location with lower Ni concentration (MI), G-nov and 4193cs increased yield by 102.59% and 114.61% in 2024 and by 50.83% and 59.91% in 2025, respectively. In the location with higher Ni (KS), compared to the control, 252 increased yield by 13.83% (in 2024) and 47.53% (in 2025). It appears that some of the rhizobial inoculants were more efficient in one, while others were more efficient in the other experimental field, adding to the complexity of analyzing the effects of inoculation on plant yield in a field experimental setting. Generally, the positive effects of inoculation on the total number of microorganisms, compared to the control, were most pronounced during the first experimental season, in autumn 2024, when the control group had lower numbers compared to all inoculated groups, when taking both fields into account. One of the indicators that rhizosphere microorganisms play an important role in alfalfa growth is the observation that TNM, together with *Azotobacter*, appeared to have a significant influence on soil respiration rate as well as alfalfa yield in the first year of the experiment. Therefore, we supposed that the increase in soil activity influenced by microorganisms could contribute to the increase in alfalfa yield (**Figure 10**). The high percentage of variation explained by our SEM model (79.9%) suggests that microbial properties and environmental factors (location and season) as well as inoculation by different strains represent a dominant mechanism explaining the alfalfa yield. The model obtained from the PLS-SEM analysis, on the other hand, emphasizes the significance of various environmental factors and rhizobial treatment that affect alfalfa yield, rather than the microbial factors present in its rhizosphere soil. Considering that alfalfa is a highly variable perennial species with multiple cuts per year, it is challenging to study the effects of different factors and identify their effects on alfalfa performances and yield (Tucak et al., 2023; Delić et al., 2010). Therefore, more research is needed for different cuts and locations under field conditions. Nevertheless, despite this limitation, the promising results obtained indicate the potential of inoculation for improving growth under stressful conditions.

## 5 Conclusion

The results of the study showed the potential of alfalfa to grow well in soils with mildly elevated Ni concentrations. The inoculation with particular rhizobia additionally improved alfalfa yield and overall microbiological properties in the alfalfa rhizosphere soils. It is found that particular strains can increase alfalfa yield up to 38% in nickel-rich soil. The total number of microorganisms, as well as other specific groups including *Azotobacter*, ammonifiers, and actinomycetes, were most sensitive to the influence of various environmental factors, including Ni concentrations, treatment, season, and their interactions, while fungi were the most stable group. This study is based on the analysis of culturable microorganisms, which may miss the contribution of uncultured functional flora. Therefore, it is necessary to comprehensively analyse the regulation of rhizobia inoculation on rhizosphere microbial functional network by combining 16S rRNA sequencing and functional gene chip technology. However, the potential application value of this study is discovering the strains of *E. meliloti* that could be used for bioremediation of Ni-burdened soils and producing higher yields of alfalfa, which could provide support for forage cultivation in heavy metal exposed areas.

## Data Availability

The original contributions presented in the study are included in the article/**Supplementary Material**. Further inquiries can be directed to the corresponding author.
