# Supplementary material for: Microbial community in alfalfa rhizosphere in response to rhizobial inoculation under increased concentrations of potentially toxic elements
Source: Front Plant Sci. 2026 Jun 16;17:1800160. doi: 10.3389/fpls.2026.1800160 (PMC13314779; doi:10.3389/fpls.2026.1800160)
Supplement: Supplementary file 1 [file DataSheet1.docx]

**Supplementary Material 1. Results of the Multivariate Tests**.

| Effect | Wilks’ lambda | F | Hypothesis df | Error df | Sig. | Partial Eta Squared |
| --- | --- | --- | --- | --- | --- | --- |
| Season | 0.009 | 59.306 | 12 | 74 | <0.001 | 0.906 |
| Location | 0.041 | 144.839 | 6 | 37 | <0.001 | 0.959 |
| Treatment | 0.007 | 9.498 | 36 | 165.239 | <0.001 | 0.560 |
| Season x Location | 0.022 | 35.261 | 12 | 74 | <0.001 | 0.851 |
| Season x Treatment | 0.001 | 7.496 | 72 | 207.106 | <0.001 | 0.687 |
| Location xTreatment | 0.007 | 9.466 | 36 | 165.239 | <0.001 | 0.559 |
| Season x Location x Treatment | 0 | 9.955 | 72 | 207.106 | <0.001 | 0.742 |

**Supplementary Material 2. Plant growth-promoting traits of the rhizobial strains used in this study.**

| Strain | IAA production (μg ml^-1^)* | Siderophore production | Phosphate solubilization (mm) | ACC deaminase | Ni biosorption | Ni tolerance (mM)* |
| --- | --- | --- | --- | --- | --- | --- |
| 218 | >200 | - | 4** | +* | +* | 1.2 |
| 224 | >200 | - | 0.5** | -* | n.d. | 1.1 |
| G-nov | >200 | - | 3 | + | + | 1.1 |
| 4193cs | >200 | - | 3** | + | +* | 1.2 |
| 217 | >200 | - | 1.5** | +* | +* | 1.1 |
| 252-345 | >200 | - | 3** | + | + | 0.7 |

PGP traits were determined using methodology according to Pešić et al. (2025) and the results marked with * derive from Pešić et al. (2025), while marked with ** from Stajković-Srbinović et al. (2012).; n.d. – not determined

**Supplementary Material 3. Shoot dry weight of alfalfa (average values for all seasons and locations).**

|  |  | Shoot dry weight (g/m^2^) |
| --- | --- | --- |
|  | 218 | 126.09 c |
|  | 224 | 139.30 bc |
| Treatment | G-nov | 172.24 a |
|  | 4193cs | 174.23 a |
|  | 217k | 155.53 ab |
|  | 252 | 159.14 ab |
|  | Control | 126.68 c |
| Source of variation | | *p* value |
| Treatment |  | 0.000 |
| Location |  | 0.420 |
| Year |  | 0.000 |
| Treatment x Location | | 0.005 |
| Treatment x Year |  | 0.020 |
| Location x Year |  | 0.000 |
| Treatment x Location x Year | | 0.433 |

Three-way ANOVA for treatment, location and year; a-c: Values in a column marked with the same letters are not statistically different according to Duncan's multiple range test (*p*˂0.05).

**Supplementary Figure 1.**


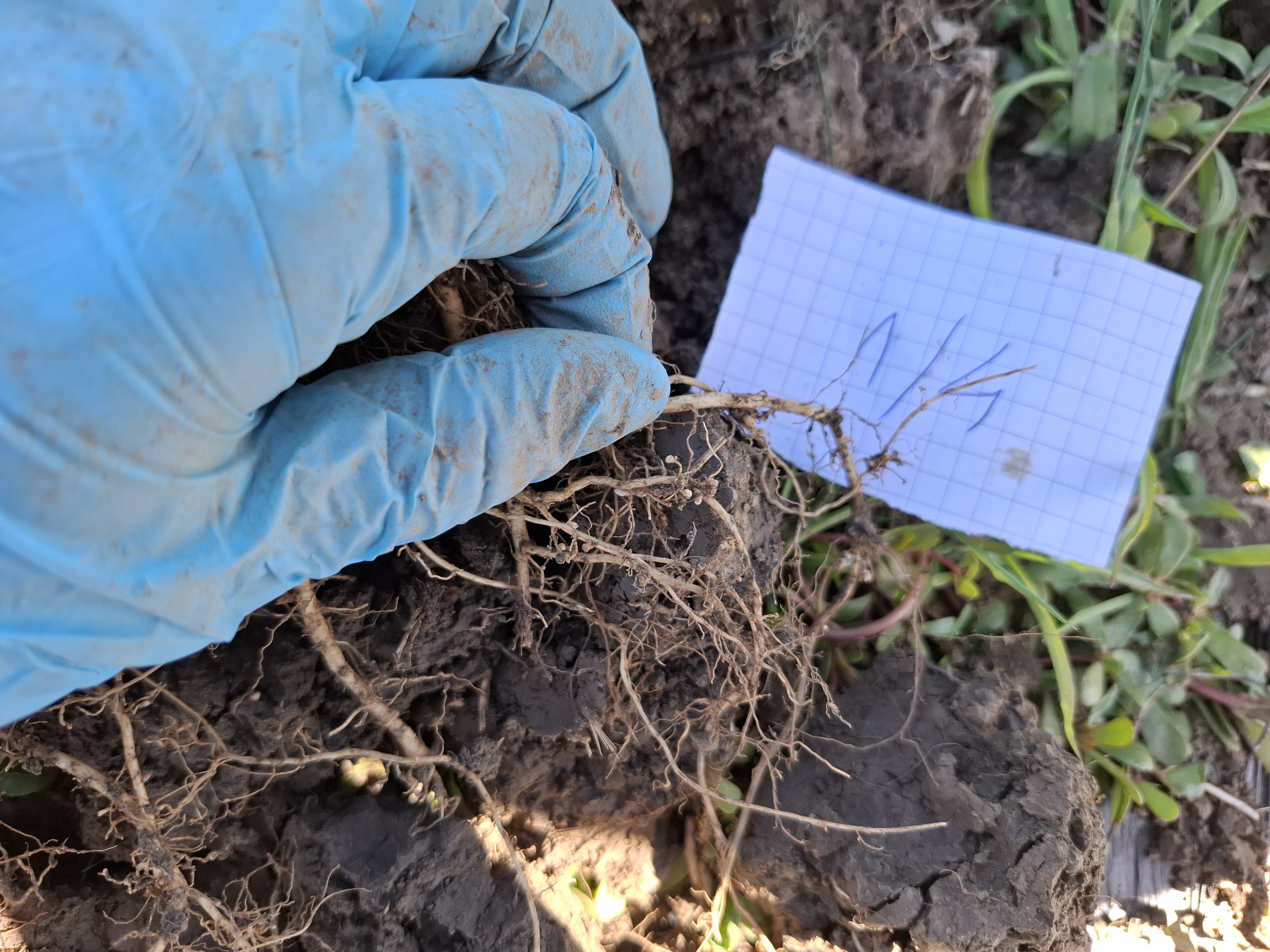


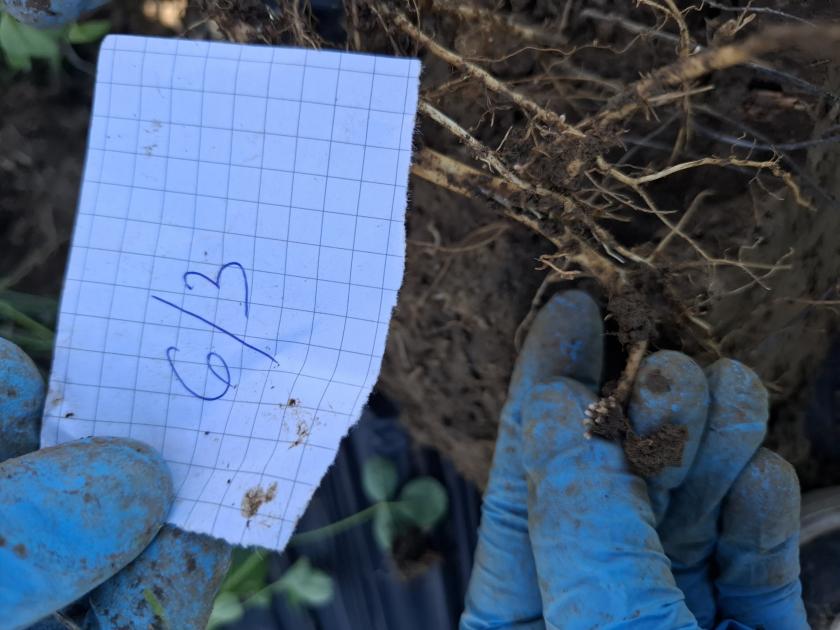

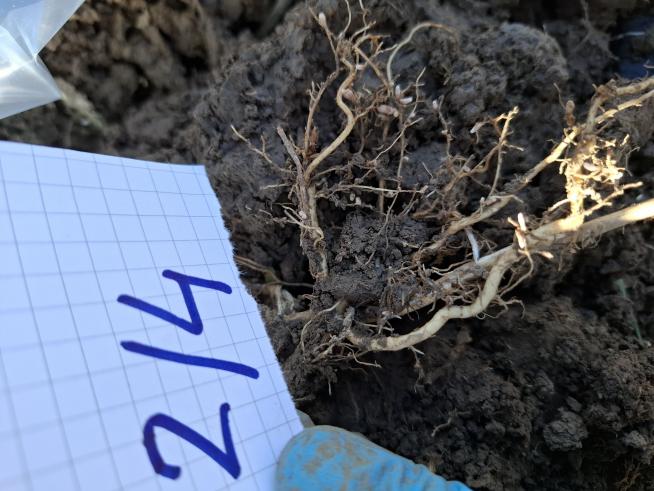


Nodules on alfalfa roots after inoculation with different *Ensifer meliloti* strains.
